# Accuracy of Vital Signs Measurements by a Smartwatch and a Portable Health Device: Validation Study

**DOI:** 10.2196/16811

**Published:** 2020-02-12

**Authors:** Christina Hahnen, Cecilia G Freeman, Nilanjan Haldar, Jacquelyn N Hamati, Dylan M Bard, Vignesh Murali, Geno J Merli, Jeffrey I Joseph, Noud van Helmond

**Affiliations:** 1 College of Medicine Radboud University Medical Center Nijmegen Netherlands; 2 Sidney Kimmel Medical College Thomas Jefferson University Philadelphia, PA United States; 3 Department of Surgery and Medicine Sidney Kimmel Medical College Thomas Jefferson University Philadelphia, PA United States; 4 Department of Anesthesiology Sidney Kimmel Medical College Thomas Jefferson University Philadelphia, PA United States; 5 Department of Anesthesiology Cooper Medical School of Rowan University Cooper University Health Care Camden, NJ United States

**Keywords:** medical devices, mHealth, vital signs, measurements validity

## Abstract

**Background:**

New consumer health devices are being developed to easily monitor multiple physiological parameters on a regular basis. Many of these vital sign measurement devices have yet to be formally studied in a clinical setting but have already spread widely throughout the consumer market.

**Objective:**

The aim of this study was to investigate the accuracy and precision of heart rate (HR), systolic blood pressure (SBP), diastolic blood pressure (DBP), and oxygen saturation (SpO_2_) measurements of 2 novel all-in-one monitoring devices, the BodiMetrics Performance Monitor and the Everlast smartwatch.

**Methods:**

We enrolled 127 patients (>18 years) from the Thomas Jefferson University Hospital Preadmission Testing Center. SBP and HR were measured by both investigational devices. In addition, the Everlast watch was utilized to measure DBP, and the BodiMetrics Performance Monitor was utilized to measure SpO_2_. After 5 min of quiet sitting, four hospital-grade standard and three investigational vital sign measurements were taken, with 60 seconds in between each measurement. The reference vital sign measurements were calculated by determining the average of the two standard measurements that bounded each investigational measurement. Using this method, we determined three comparison pairs for each investigational device in each subject. After excluding data from 42 individuals because of excessive variation in sequential standard measurements per prespecified dropping rules, data from 85 subjects were used for final analysis.

**Results:**

Of 85 participants, 36 (42%) were women, and the mean age was 53 (SD 21) years. The accuracy guidelines were only met for the HR measurements in both devices. SBP measurements deviated 16.9 (SD 13.5) mm Hg and 5.3 (SD 4.7) mm Hg from the reference values for the Everlast and BodiMetrics devices, respectively. The mean absolute difference in DBP measurements for the Everlast smartwatch was 8.3 (SD 6.1) mm Hg. The mean absolute difference between BodiMetrics and reference SpO_2_ measurements was 3.02%.

**Conclusions:**

Both devices we investigated met accuracy guidelines for HR measurements, but they failed to meet the predefined accuracy guidelines for other vital sign measurements. Continued sale of consumer physiological monitors without prior validation and approval procedures is a public health concern.

## Introduction

### Background

In recent years, advances in technology and the availability of ample venture capital have been combining to produce a growing array of new medical diagnostic devices. New consumer devices are being developed to easily monitor multiple physiological parameters at home or on the go—often connecting with mobile devices to provide user-friendly updates of health status (mobile health). The vision behind these devices is that they will transform conventional medicine into *digital medicine*, facilitating a transition from treating disease to promoting health, from being reactive to being proactive, from being general to being individualized, from offering office-based health care to bringing health care to patients, and from interrupting daily life to being incorporated into it [1].

This vision is appealing, but presently, some of the publicized work in the field of consumer physiological monitoring appears to be characterized by excessive hype [2]. Many of these new technologies have yet to be formally studied in a clinical setting, and there are more than a few examples of digital *snake oil* [2] with substantial societal uptake of devices before their eventual discrediting [3]. This practice appears to be a barrier to truly advancing the field of consumer physiological monitoring.

Smartwatches are one type of consumer device to easily monitor physiological parameters on a regular basis, and more recently, *medical tricorders* have been introduced. A medical tricorder [4] is an all-in-one handheld portable device to be used by consumers to quickly obtain several vital sign measurements to monitor medical conditions.

### Objectives

The aim of this study was to assess the accuracy of vital signs measurements by 2 novel all-in-one physiological monitoring devices, a smartwatch, and a medical tricorder.

## Methods

### Ethical Approval

This study was approved by the Institutional Review Board of Thomas Jefferson University (IRB-nr: 18D.358), and subjects were enrolled between June 27, 2018 and November 9, 2018. Before participation, all subjects provided written informed consent after all procedures and study risks were fully explained.

### BodiMetrics Performance Monitor

**Figure 1:**
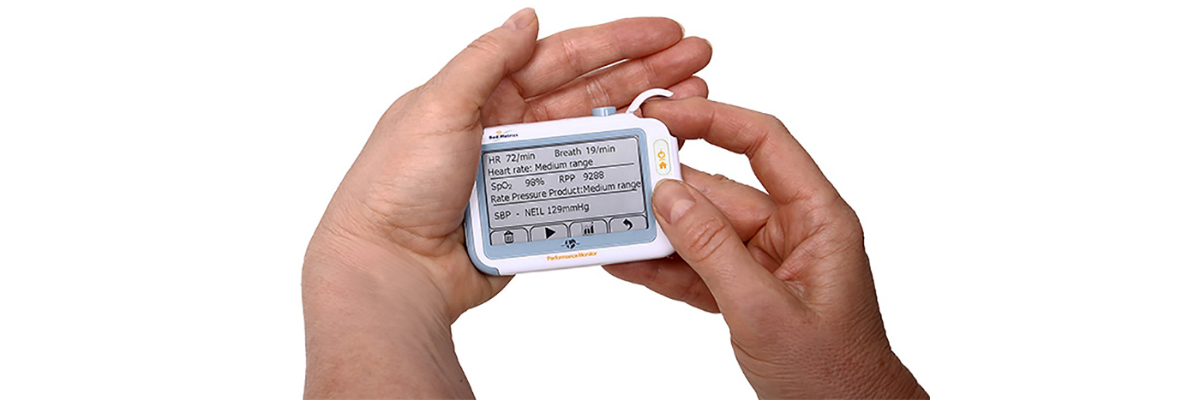
BodiMetrics Performance Monitor tricorder. Vital sign measurements are performed by placing the right index finger on the plethysmography sensor in the right upper corner under the flap. In addition, contact has to be made with the electrocardiogram electrodes at the front, left lateral side, and back using both hands.

The BodiMetrics Performance Monitor (BodiMetrics, Manhattan Beach) is a commercially available tricorder that is sold by several major US-based retailers such as Walmart, Amazon, and Costco. Due to its pocket-size (88×56×13 mm), it can easily be carried around for frequent measurement of vital signs. To create a user profile, it requires the input of sex, date of birth, height and weight, and an initial calibration for systolic blood pressure (SBP) obtained with a conventional upper-arm sphygmomanometer. The tricorder provides measurements of SBP, oxygen saturation (SpO_2_), and heart rate (HR) via different sensors, and the measurements are displayed on a touch screen. The device uses audio and visual instructions to guide users through a measurement; the right index finger needs to be placed beneath the cap on top, the right thumb on the electrode on the front, and the right middle finger on the electrode on the back of the device (Figure 1). Then, the electrode on the left lateral side needs to be placed in the left palm. To ensure a successful measurement, contact needs to be maintained with all electrodes, while the index finger is inserted under the cap. A measurement takes about 30 seconds to complete. HR is measured through contact with the electrodes, whereas SpO_2_ is measured using a plethysmography sensor under the top flap. SBP is measured through the determination of pulse transit time from the electrocardiogram (ECG) and photoplethysmography signals [5]. According to the manufacturer specifications, HR can be measured between 30 to 210 bpm and SpO_2_ can be measured between 70% and 100% [6]. The manufacturer does not provide information about the SBP measurement range.

### Everlast TR10 Smartwatch

**Figure 2:**
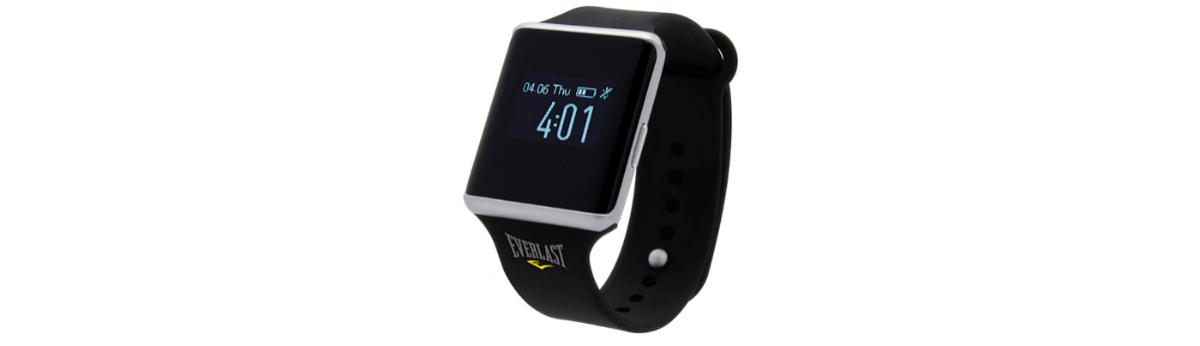
Everlast smartwatch. To enable a physiological measurement, the watch must be worn on the bare wrist making contact with the skin. Measurements are initiated by pressing the button on the right side of the watch.

The Everlast TR10 smartwatch (Figure 2; Everlast) is a smartwatch that is for sale through several US-based retailers such as Walmart and Amazon. Unlike the BodiMetrics tricorder, the Everlast smartwatch does not require any user specific information or a calibration before use. It provides measurements of SBP, diastolic blood pressure (DBP), and HR. Results are shown on a display, and a button on the side is used to navigate through the different measurements. We were unable to verify the underlying measurement methods with the manufacturer. The back plate of the watch contains contact electrodes and a photoplethysmography sensor, which we presume are utilized for the different physiological measurements.

### Standard Device

We used the validated Cardiocap/5 (Datex-Ohmeda) hospital-grade vital signs monitor for reference measurements [7]. The Cardiocap/5 has a mean blood pressure (BP) measurement range of 25 to 260 mm Hg in adults. It uses a plethysmography sensor to measure SpO_2_. HR can be measured using ECG or can be derived from the SpO_2_ measurement. The measurement range for peripheral SpO_2_ is 40% to 100% and 30 to 250 bpm for HR. The measurement accuracy for SpO_2_ between 80% and 100% is ±2% and between 50% and 80% is ±3%. The accuracy for HR is ±5% or ±5 bpm depending on which of the two is greater [8]. The manufacturer does not provide any information about the BP accuracy, but the device does fulfill the American National Standards Institute (ANSI)/Association for the Advancement of Medical Instrumentation (AAMI)/International Organization for Standardization (ISO) guidelines. This means that its measurements are accurate within 5 mm Hg with a SD of ≤8 mm Hg [9,10]. To confirm the Cardiocap’s accuracy in BP measurements, we compared Cardiocap noninvasive BP measurements with intra-arterial BP measurements from a previously published dataset [11]. The average absolute difference between the two methods from six paired BP measurements in 100 subjects (582 total pairs) was 4.3 (SD 6.8) mm Hg for SBP and 4.8 (SD 7.2) mm Hg for DBP measurements. This difference is within the recommended limits for accuracy when comparing the obtained measurements from a noninvasive monitor with intra-arterial measurements [9,10].

For BP measurements, the participant’s arm circumference was measured, and the appropriate cuff size was chosen accordingly. The standard adult-size cuff (REF572428) and the large adult-size cuff (REF 572429, both Datex-Ohmeda, Inc) were used for arm circumferences of 25 to 35 cm and 33 to 47 cm, respectively.

### Subjects

We recruited study participants, aged more than 18 years, from patients visiting the Thomas Jefferson University Hospital Preadmission Testing Center. Exclusion criteria were as follows: (1) contraindication for automated BP measurement on both arms for reasons including but not limited to a history of breast cancer surgery with radiation therapy or axillary lymph node dissection, arteriovenous fistula for hemodialysis, or an open wound; (2) irregular heart rhythms such as atrial fibrillation and atrial flutter; (3) missing upper extremity, hand, or finger; (4) inability to wear a watch because of wrist circumference or edema of the arm, wrist, or hand; (5) lack of appropriate-sized BP cuff; and (6) pacemaker or other implanted medical device [6].

### Testing Procedure

**Figure 3:**
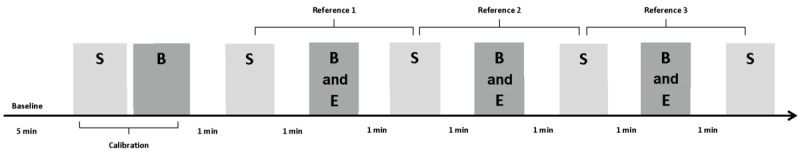
Study timeline. B: BodiMetrics Performance Monitor measurement; E: Everlast smartwatch measurement; S: Standard measurement (light grey color). The dark grey color indicates the investigational devices.

Research staff were trained to measure BP, SpO_2_, and HR with the Everlast smartwatch, BodiMetrics tricorder, and Cardiocap/5 according to their manufacturers’ guidelines. The investigational devices used in the study were new devices and were acquired through Amazon shortly before the study commenced. We followed a validation protocol derived from the ANSI/AAMI/ISO 2013 standards for evaluating noninvasive automated sphygmomanometers [3,9,10]. Study procedures were explained, and participants were seated in a chair with back support and armrests, with both feet on the floor; subjects were instructed not to cross their legs or speak during the study. After 5 min of rest, the measurement protocol began with an initial standard measurement and a calibration measurement for the BodiMetrics tricorder (Figure 3). After this calibration, sequential measurements were taken, alternating between the reference and the investigational devices, with 60 seconds in between each measurement. This yielded a pattern where two standard measurements bounded each investigational device measurement [9,10]. In total, four standard and three investigational device measurements were obtained per participant. If a measurement with one of the investigational devices failed, up to two additional attempts were made. Participants were blinded to the standard measurements but not to the investigational measurements as these required the participants’ interaction with the devices.

### Data and Statistical Analysis

The reference SBP, DBP, HR, and SpO_2_ values were all calculated by determining the average of the two standard vital sign measurements that bounded the investigational measurements (Figure 3). This yielded three reference-investigational comparison pairs for the different vital signs for each device. As our protocol was derived from a BP validation protocol, we excluded data from subjects with a variation in standard measurements greater than 12 mm Hg for SBP and 8 mm Hg for DBP, in accordance with validation guidelines [9,10].

For BP measurement validation, the main outcome was the mean (SD) of the absolute difference between the respective investigational devices and the reference values for SBP and DBP [9,10]. The BP measurement results have been presented elsewhere previously [12] and are reported again with permission. BP measurements by the investigational devices were considered accurate if the mean absolute difference was ≤5 mm Hg with a SD of ≤8 mm Hg [9,10]. Accuracy of the BP measurements by the investigational devices was also graded according to the classification from the British Society of Hypertension [13,14].

For HR measurement validation, the main outcomes were the mean of the absolute difference between the respective investigational devices and the reference values, and the percent absolute difference between the respective investigational devices and the reference values. HR measurements were considered accurate if the mean absolute difference was within either ±10% or ±5 bpm, depending on which of the two was greater [15].

For SpO_2_, the main outcome was the root mean square error (RMSE) between the respective investigational devices and the reference values. SpO_2_ measurements were considered accurate if the mean RMSE was ≤3.0% [16].

The main outcome data were visualized using Bland-Altman plots (Sigmaplot, version 14, Systat Software Inc). The dotted line in the Bland-Altman plot represents the mean relative difference (investigational minus reference), and the dashed lines represent ±1.96 SDs for the absolute difference. In addition, correlation analyses and scatterplots were utilized to assess the relation between the respective investigational devices and the reference values. To aid in the interpretation of clinical applicability of these devices, we also assessed the rates at which they successfully detected values for vital signs that were measured outside the normal range by reference values (≥140 mm Hg SBP, ≥90 mm Hg DBP, <60 bpm HR, or <90% for SpO_2_). The solid line in the scatterplots represents the line of identity. The dashed lines in the scatterplots for BP, HR, and SpO_2_ represent the cutoff for stage 2 hypertension, bradycardia, and hypoxemia, respectively. Normality of values was assessed using the Shapiro-Wilk test. In the case of normally distributed residuals, Pearson correlation analysis was performed, and in the case of non-normally distributed residuals, Spearman correlation analysis was performed. Means are reported with SD for all variables. Nominal variables are reported as n with relative proportion in percentage.

All data files are available from the Data Archiving and Networked Services database [17].

## Results

### Principal Results

We enrolled a total of 127 subjects, and data from 41 participants were discarded because of excessive variation in sequential standard BP measurements, as specified by the ANSI/AAMI/ISO 2013 standards for evaluating noninvasive automated sphygmomanometers [3,9,10], and from 1 participant because of repeated failure of BodiMetrics calibration; 85 subjects were included in the final analysis. Demographics and characteristics of the study population are displayed in Table 1. These data have previously been presented by Van Helmond et al [12] and are reproduced with permission.

**Table 1:** Validation study: participant characteristics (n=85).

Participant characteristics				Values
Systolic blood pressure at baseline (mm Hg), mean (SD)				125 (15)
Diastolic blood pressure at baseline (mm Hg), mean (SD)				76 (9)
Heart rate at baseline (bpm), mean (SD)				72 (12)
Oxygen saturation at baseline (%), mean (SD)				96 (2)
Age (years), mean (SD)				53 (21)
Body mass index (kg/m^2^), mean (SD)				28 (7)
**Gender, n (%)**				
	Male		49 (58)	
	Female		36 (42)	
**Ethnicity, n (%)**				
	White		61 (72)	
	Black		12 (14)	
	Asian		6 (7)	
**Education, n (%)**				
	High school or General Educational Diploma		28 (33)	
	College or university degree		38 (45)	
	Master’s degree		6 (7)	
	Doctorate		5 (6)	
**Self-reported medical history, n (%)**				
	**Hypertension**		32 (38)	
		Taking medication for hypertension	31 (36)	
	**Diabetes**		13 (15)	
		Taking medication for diabetes	12 (14)	
	**Heart attack**		3 (4)	
		Taking medication for heart attack	3 (4)	
	**Heart failure**		2 (2)	
		Taking medication for heart failure	1 (1)	
	**Peripheral vascular disease**		2 (2)	
		Taking medication for peripheral vascular disease	2 (2)	
	**Stroke**		1 (1)	
		Taking medication for stroke	0 (0)	
	Smoking		8 (9)	

### BodiMetrics Performance Monitor

#### Blood Pressure

**Figure 4:**
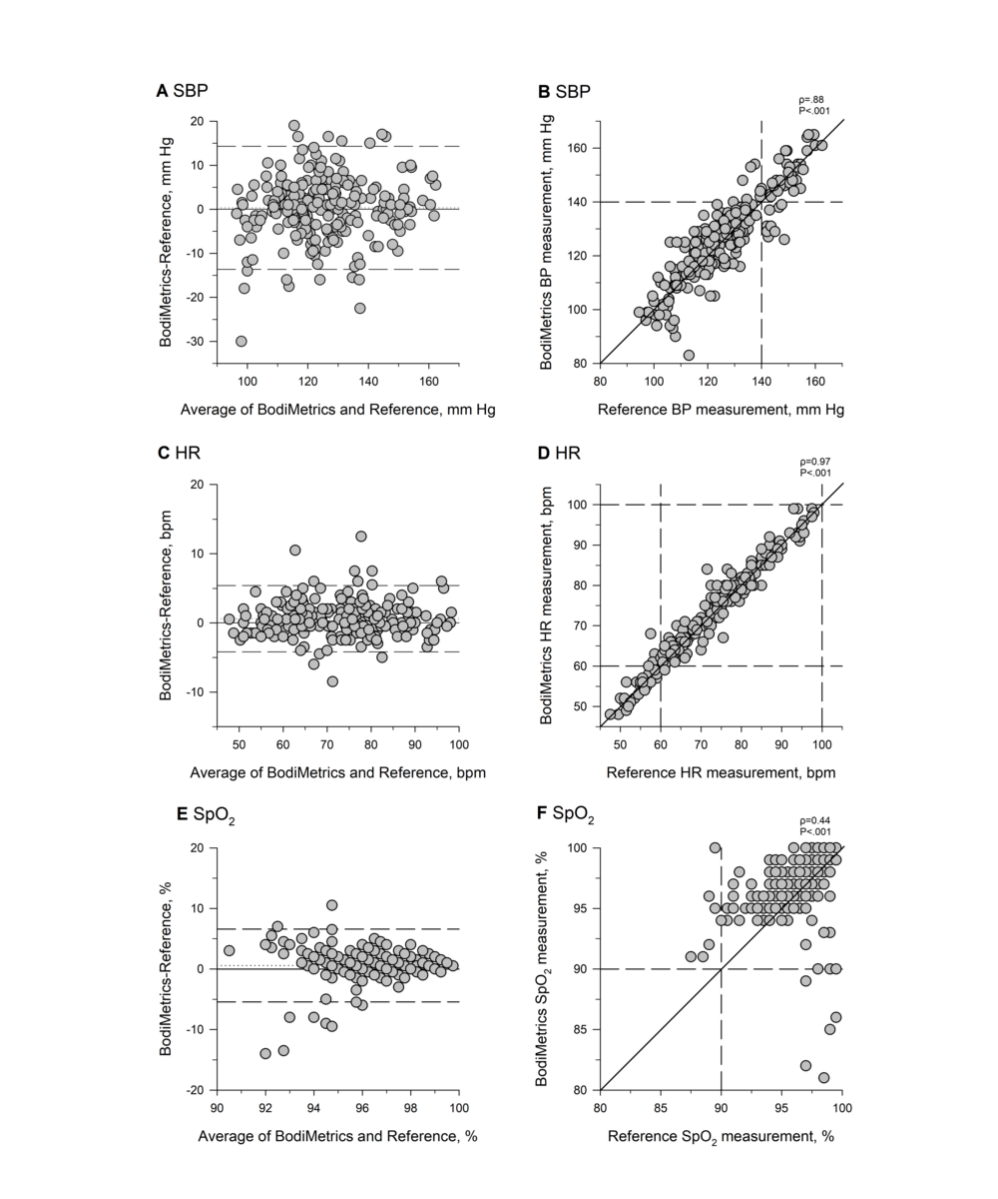
Systolic blood pressure (A and B), heart rate (C and D), and oxygen saturation (E and F) measurements by BodiMetrics tricorder and reference values. SBP: systolic blood pressure, HR: heart rate, SpO_2_: oxygen saturation.

The BodiMetrics tricorder failed in 6 (7%) participants for a total of 13 (5%) of the maximum 255 BP measurements that could have been obtained in the 85 participants. The average absolute difference between the BodiMetrics tricorder and the reference was 5.3 (SD 4.7) mm Hg for SBP (Figure 4). The performance monitor, thus, failed to meet the predefined accuracy target for SBP measurements [9,10]. According to the British Society of Hypertension guidelines, the BodiMetrics is a grade-B BP monitor [13,14]. BodiMetrics tricorder measurements correlated well with reference measurements (ρ=0.88; *P*<.001); the BodiMetrics tricorder measured a hypertensive B*P* value (≥140 mm Hg) for 80% of the hypertensive reference SB*P* values (Figure 4).

#### Heart Rate

The BodiMetrics tricorder failed in 3% (3/85) participants for a total of 3.1% (8/251) HR measurements. The average absolute difference between the BodiMetrics tricorder and the reference values was 1.8 (SD 1.8) bpm (Figure 4). The mean absolute percentage difference was 2.5 (SD 2.5) %. The BodiMetrics tricorder, therefore, met the predefined accuracy cutoff for HR measurements [15]. Correlation analysis revealed a statistically significant, strong correlation (ρ=0.97; *P*<.001; Figure 4) between the BodiMetrics tricorder HR measurements and the reference values. The BodiMetrics tricorder measured a bradycardic HR value (<60 bpm) for 90% of the bradycardic reference HR measurements (Figure 4).

#### Oxygen Saturation

The BodiMetrics tricorder failed in 4% (4/85) participants for a total of 3.5% (9/257) SpO_2_ measurements. The RMSE for SpO_2_ between BodiMetrics Performance Monitor measurements and reference values was 3.1% (Figure 4). The BodiMetrics Performance Monitor measurements, thus, failed to meet the predefined accuracy standard [16]. BodiMetrics Performance Monitor SpO_2_ measurements were moderately correlated with reference values (ρ=0.44; *P*<.001; Figure 4). The BodiMetrics tricorder measured no hypoxic SpO_2_ values (<90%) for any of the hypoxic reference SpO_2_ measurements (Figure 4).

### Everlast TR10 Smartwatch

#### Blood Pressure

**Figure 5:**
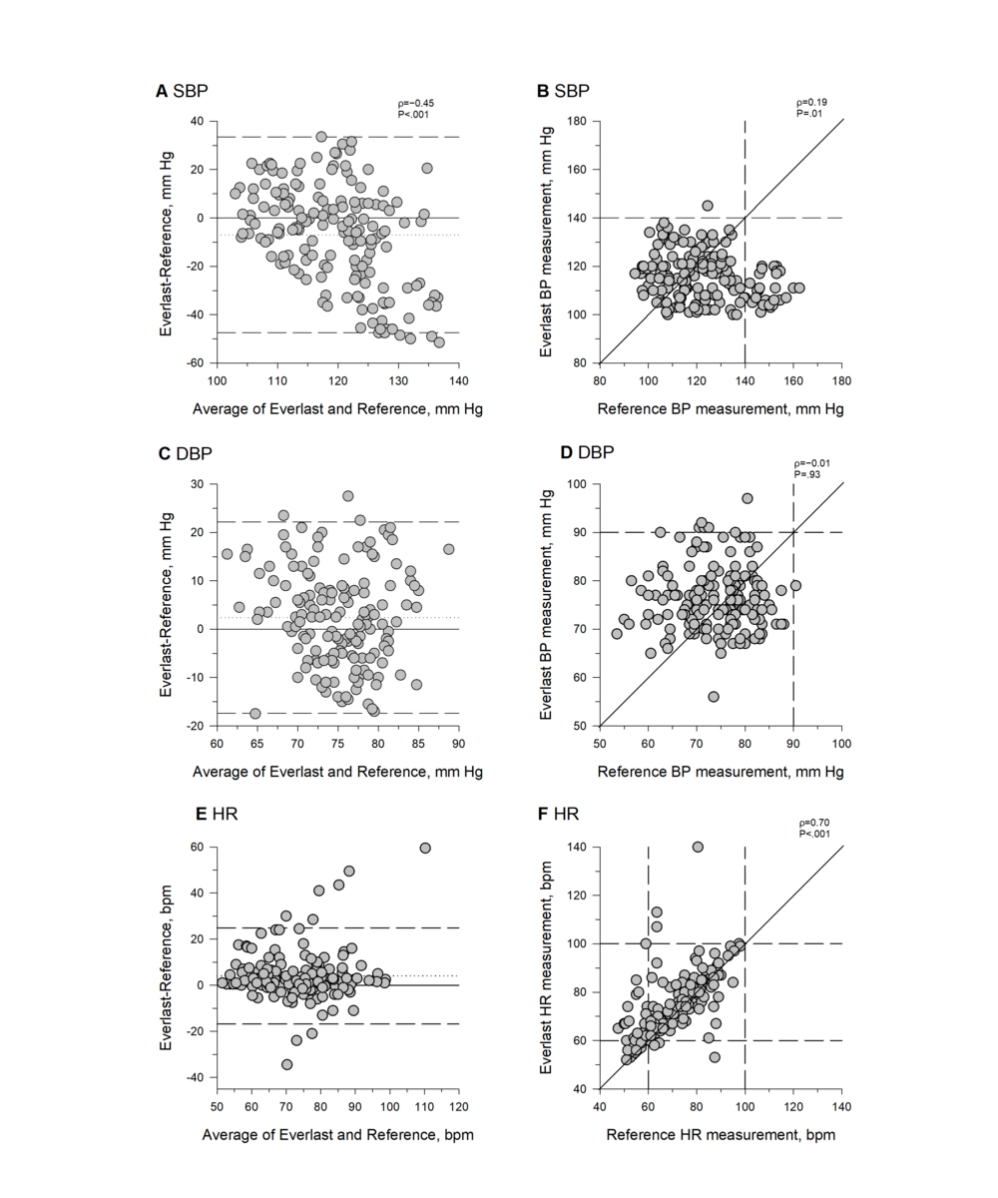
Systolic blood pressure (A and B), diastolic blood pressure (C and D), and heart rate (E and F) measurements by Everlast smartwatch and reference values. BP: systolic blood pressure, DBP: diastolic blood pressure, HR: heart rate.

The Everlast watch failed in 38% (33/85) participants for a total of 34.1% (87/255) BP measurements. The average absolute differences between the Everlast watch and reference were 16.9 (SD 13.5) mm Hg for SBP and 8.3 (SD 6.1) mm Hg for DBP (Figure 5). The watch’s performance, thus, failed to meet the predefined accuracy guideline for SBP and DBP measurements and is considered a grade-D monitor for SBP and DBP measurements according to the British Society of Hypertension guidelines [9,13,14]. The difference between the Everlast watch and reference measurement was dependent on the SB*P* value, such that lower SBPs were estimated higher and higher SBPs were estimated lower (ρ=−0.45; *P*<.001; Figure 5). Everlast BP measurements were not correlated with reference BP measurements, and the Everlast watch failed to measure any hypertensive B*P* values for any of the hypertensive reference SBP or DBP measurements (Figure 5).

#### Heart Rate

The Everlast watch failed in 36% (31/85) participants for a total of 31.8% (81/255) HR measurements. The average absolute difference between the Everlast watch and the reference was 6.5 (SD 9.2) bpm (Figure 5). The mean absolute percentage difference was 9.9 (SD 14.3) %. The Everlast watch, therefore, met the predefined accuracy guidelines [15]. Correlation analysis revealed a significant moderate correlation (ρ=0.7; *P*<.001) between the Everlast watch HR measurements and the reference values (Figure 5). The Everlast smartwatch measured a bradycardic HR value (<60 bpm) for 33% of the bradycardic reference HR measurements (Figure 5).

## Discussion

### Principal Findings

The aim of this study was to assess the accuracy of vital sign measurements by 2 novel, all-in-one physiological monitoring devices, a smartwatch, and a medical tricorder. We found that the accuracy guidelines for HR measurements were met by both investigational devices. However, neither device met the accuracy guidelines for BP measurements. The SpO_2_ measurements by the BodiMetrics also did not meet the accuracy guidelines for transmissive pulse oximetry. The absolute or relative differences from the reference measurements were very large for the Everlast watch, whereas the BodiMetrics’ measurements were closer to meeting the predefined standards.

The results of our study indicate that the Everlast smartwatch is not accurate enough to be used to monitor vital signs. For the BP measurements, the Everlast smartwatch did not correctly measure any (0%) of the hypertensive values for the values that were hypertensive when measured with the standard cuff. Although the watch met our predefined accuracy standard for HR, it detected only 33% of the bradycardic HR values that were measured by the standard monitor. In addition to the accuracy problems, the watch failed to obtain any measurement at all for 32% of HR and 34% of SBP and DBP measurements.

We are not aware of any prior studies on the Everlast smartwatch to compare the findings of this study with, but we found 3 studies by a Dutch research group on the BodiMetrics Performance Monitor, which is marketed in Europe under the name Checkme [5,18,19]. A study by Schoot et al [5] compared the SBP measurements obtained with the BodiMetrics Performance Monitor with the SBP measurements obtained by a reference automated cuff in 37 outpatients in supine position and sitting position and found average absolute differences of approximately 6.7 (SD 5.4) mm Hg in supine position and approximately 10.1 (SD 7.0) mm Hg in sitting position. The average absolute difference they found is somewhat larger than the difference we found in this study (5.3 [SD 4.7] mm Hg). An underlying reason for this difference may be that the bias calculation in their study did not average the two standard measurements that bounded each investigational measurement, and that naturally occurring drift in BP, thus, may have exaggerated the detected difference [9]. They also did not exclude any subjects based on drift in standard measurements, as we did per the ANSI/AAMI/ISO BP monitor validation protocol [9]. In a subsequent study by Weenk et al [18], the same group compared all vital signs measured by the BodiMetrics with a standard hospital-grade monitor in 41 Internal Medicine inpatients. They found an average absolute difference of 10.7 (SD 11.0) mm Hg between BodiMetrics SBP measurements and the reference. For HR, the average absolute difference was 2.9 (SD 2.9) bpm, and for SpO_2_, the RMSE was 4.2% [18]. These BodiMetrics-to-reference differences are substantially greater than the difference we encountered and are potentially because of a smaller dataset of 69 data pairs per vital sign compared with our 242 BP, 246 SpO_2_, and 247 HR data pairs. There were also differences in the measurement protocol between the Weenk et al’s study [18] and this study. They obtained data from inpatients in supine position, whereas our subjects were seated outpatients. Moreover, we averaged bounding standard measurements to reduce the influence of drift, whereas Weenk et al [18] did not. Another difference is that they performed calibration of the BodiMetrics once in the morning and then collected data on three different time points during the day, whereas our measurements were taken in the approximately 20 to 30 min following calibration [18]. The subject’s BP may have changed significantly from the value at which the BodiMetrics was calibrated in their study, which might have affected the accuracy of the measurements. In contrast, the BP during our protocol was likely similar to the calibration value [20]. In a third study from the same group, Ogink et al [19] compared BodiMetrics SBP measurements with SBP measurements obtained at home by 11 patients with hypertension using various home automated BP monitors over 3 weeks. BodiMetrics SBP measurements were found to be weakly correlated to cuff SBP measurements, and there was a large absolute difference between the two measurements (eg, 44% of measurements differed by >10 mm Hg). Although the accuracy of the BodiMetrics is difficult to assess from this study, considering there was no standardized monitor or measurement protocol, the reported low accuracy appears to indicate that the BodiMetrics SBP measurement becomes significantly more inaccurate when some time passes since calibration.

To address concerns related to BP calibration dependency, an alternative validation protocol specific to cuff-less monitors has been suggested by the Institute of Electrical and Electronics Engineers (IEEE). This validation protocol requires the same accuracy as the ANSI/AAMI/ISO standard for cuff-based devices, but it differs from the ANSI/AAMI/ISO protocol, in that it includes validation measurements after artificial changes in BP are induced after initial calibration to ensure accuracy over a wide range of B*P* values. In addition, the IEEE protocol includes validation measurements obtained after a significant period (weeks to months) since the initial calibration to investigate time-dependent calibration integrity [20]. We did not induce different BPs or investigate time-dependent changes in accuracy in this study.

**Figure 6:**
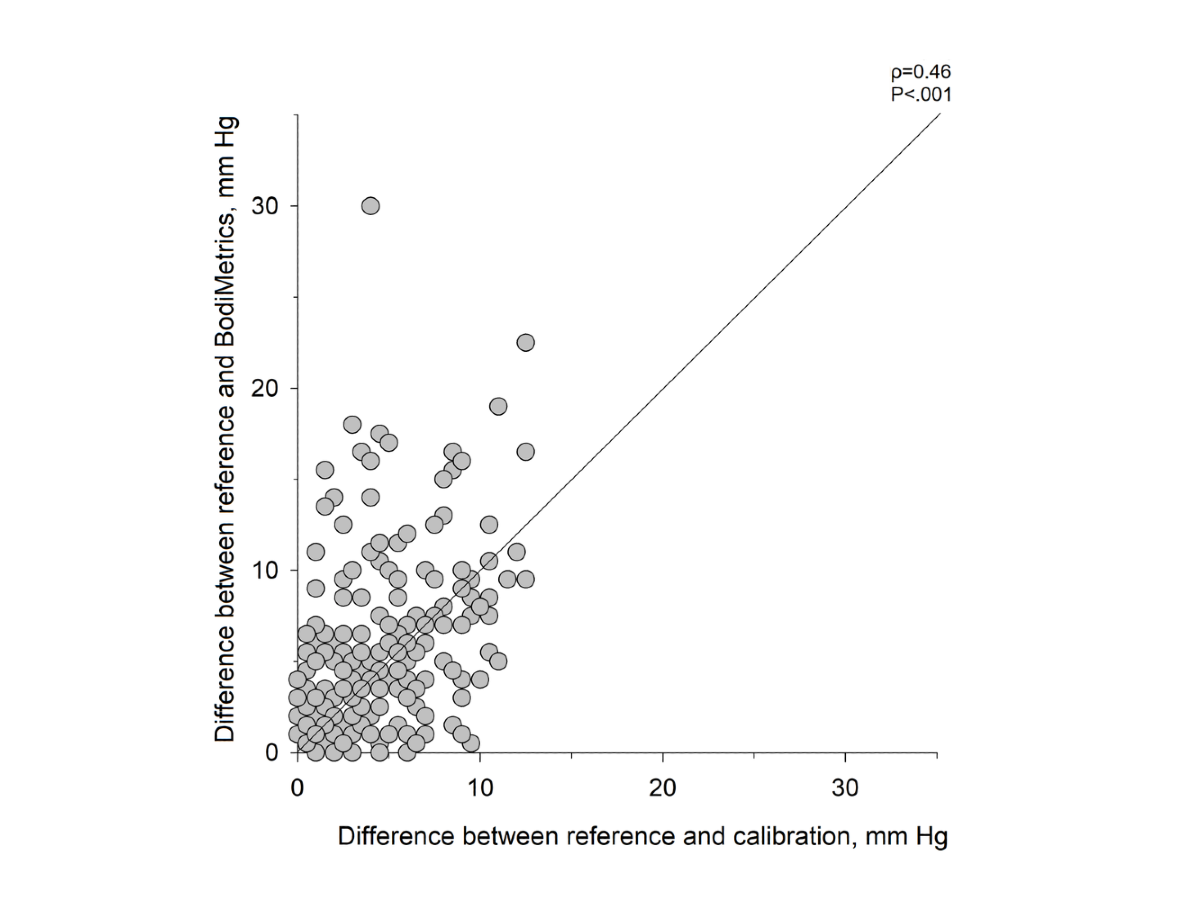
Difference between BodiMetrics tricorder systolic blood pressure measurements and calibration measurement versus difference between reference systolic blood pressure measurements and calibration measurement. The solid line in the scatterplot represents the line of identity. Data shown were not normally distributed (Shapiro-Wilk test).

To assess whether the BodiMetrics accuracy may be affected by changes in BP from the calibration value, we performed a posthoc analysis on the difference between the reference values and the BodiMetrics SBP measurements versus the difference in the reference values and the calibration value (Figure 6). We found a significant moderate correlation between these two absolute differences, indicating that the accuracy of the BodiMetrics tricorder incrementally decreases when it is used at incrementally different pressures from the calibration value. These findings would need to be confirmed in a prospective manner while consciously changing BP in study subjects to warrant any definitive conclusions. On the basis of our findings, we conclude that the current calibration process demonstrates a limitation of the BodiMetrics tricorder that should be further examined.

With regard to the initial calibration of the BodiMetrics, Weenk et al [18] reported that in 18% of the participants the calibration procedure failed and that the main reasons for failed calibration were shivering and cold hands. In the study conducted by Schoot et al [5], 12 of the 52 (23%) volunteers were excluded because of repeated calibration failure. We started our study during the hot summer months and only recognized a correlation between calibration issues and cold hands in 1 participant who was tested in late October. Overall, our calibration failure rate was lower than that reported by Weenk et al [18] who conducted their study between March and May [18]. As reported in the BodiMetrics’ users guide, dry and cold hands can influence the connectivity between hands and electrodes [5]. The conductivity is also affected by a thick stratum corneum [21]. The study by Weenk et al [18] found no correlation between patient gender, age, or weight and failure of calibration. As we only observed a failure in calibration at first or second attempt in 4.7% of attempts, we were not able to study any of these relationships.

### Practical Implications and Future Directions

Use of devices such as the Everlast smartwatch may result in individuals incorrectly assuming that they are, for example, normotensive or hypertensive. This might delay diagnosis or result in incorrect medication self-adjustments. The BodiMetrics tricorder’s accuracy for SBP and HR was considerably better than the accuracy of the Everlast smartwatch in this study. However, the BodiMetrics tricorder did not meet the predefined accuracy standards for SBP and SpO_2_ measurements. The BodiMetrics tricorder has approval for measurements of SpO_2_ and HR from the US Food and Drug Administration, but not for measurement of SBP [22]. The results of this study suggest that it is doubtful that the tricorder should be used for SBP or SpO_2_ measurements. Proper validation of consumer vital sign monitors before commercial release would aid in avoiding the potential serious repercussions of inaccurate vital sign measurements.

### Limitations

A limitation that pertains to this study is that we modified the BP monitor validation protocol that our study was based on by using an automated hospital vital signs monitor instead of a mercury sphygmomanometer [14] and auscultation. We made this adjustment to accommodate the assessment of different vital signs in one protocol and this adjustment is based on the precedent that other groups have set by following a similar approach [3,5,18,19]. In the near feature, we aim to conduct a study on cuff-less BP monitors using a mercury sphygmomanometer as a reference.

Another area of interest for future studies is the accuracy and precision of consumer vital sign monitors in real-world settings by individuals in their home environment, as that is where these devices are ultimately being used [19]. Such studies could also address whether issues related to maintenance, servicing, and wear and tear of devices adversely affect performance.

### Conclusions

The Everlast TR10 smartwatch is not accurate enough to be used as a vital sign’s measurement device. The BodiMetrics device was substantially more accurate, but it still failed to meet predefined accuracy guidelines for SBP and SpO_2_. The continued sale of consumer physiological monitor devices without the required prior validation and market approval procedures is a significant public health concern.

## Abbreviations

AAMI: Association for the Advancement of Medical Instrumentation
ANSI: American National Standards Institute
BP: blood pressure
DBP: diastolic blood pressure
ECG: electrocardiogram
HR: heart rate
IEEE: Institute of Electrical and Electronics Engineers
ISO: International Organization for Standardization
RMSE: root mean square error
SBP: systolic blood pressure
SpO_2_: oxygen saturation

## References

[1] Torkamani A, Andersen KG, Steinhubl SR, Topol EJ (2017). High-definition medicine. Cell.

[2] Klasko SK, Chen AF (2016). Digital 'Snake Oil' or the future of healthcare?. Healthc Transform.

[3] Plante TB, Urrea B, MacFarlane ZT, Blumenthal RS, Miller ER, Appel LJ, Martin SS (2016). Validation of the instant blood pressure smartphone app. JAMA Intern Med.

[4] Roddenbery G Goodreads.

[5] Datex-Ohmeda, Inc (2007). Biomedical Manuals.

[6] (2016). BodiMetrics.

[7] US Food & Drug Administration (2001). Food and Drug Administration.

[8] Pacagnella RC, Souza JP, Durocher J, Perel P, Blum J, Winikoff B, Gülmezoglu AM (2013). A systematic review of the relationship between blood loss and clinical signs. PLoS One.

[9] Graves J, Quinn D (2013). Non-Invasive Sphygmomanometers — Part 2: Clinical Investigation of Automated Measurement Type.

[10] Stergiou GS, Alpert BS, Mieke S, Wang J, O'Brien E (2018). Validation protocols for blood pressure measuring devices in the 21st century. J Clin Hypertens (Greenwich).

[11] Sheshadri V, Tiwari AK, Nagappa M, Venkatraghavan L (2017). Accuracy in blood pressure monitoring: the effect of noninvasive blood pressure cuff inflation on intra-arterial blood pressure values. Anesth Essays Res.

[12] van Helmond N, Freeman CG, Hahnen C, Haldar N, Hamati JN, Bard DM, Murali V, Merli GJ, Joseph JI (2019). The accuracy of blood pressure measurement by a smartwatch and a portable health device. Hosp Pract (1995).

[13] O'Brien E, Waeber B, Parati G, Staessen J, Myers M G (2001). Blood pressure measuring devices: recommendations of the European Society of Hypertension. BMJ.

[14] O'Brien E, Petrie J, Littler W, de Swiet M, Padfield PL, Altman DG, Bland M, Coats A, Atkins N (1993). An outline of the revised British Hypertension Society protocol for the evaluation of blood pressure measuring devices. J Hypertens.

[15] ANSI/AAMI (2002). Cardiac Monitors, Heart Rate Meters, and Alarms.

[16] Center for Devices and Radiological Health (2013). Food and Drug Administration.

[17] van Helmond N (2019). DANS easy - KNAW (Data Archiving and Networked Services).

[18] Weenk M, van Goor H, van Acht M, Engelen LJ, van de Belt TH, Bredie SJ (2018). A smart all-in-one device to measure vital signs in admitted patients. PLoS One.

[19] Ogink PA, de Jong JM, Koeneman M, Weenk M, Engelen LJ, van Goor H, van de Belt TH, Bredie SJ (2019). Feasibility of a new cuffless device for ambulatory blood pressure measurement in patients with hypertension: mixed methods study. J Med Internet Res.

[20] IEEE (2014). Engineering in Medicine and Biology Society - IEEE Standard for Wearable, Cuffless, Blood Pressure Measuring Devices.

[21] (2016). BodiMetrics.

[22] (2015). US Food & Drug Administration.

